# The Mechanism of Stem Cell Aging

**DOI:** 10.1007/s12015-021-10317-5

**Published:** 2022-01-09

**Authors:** Liangyu Mi, Junping Hu, Na Li, Jinfang Gao, Rongxiu Huo, Xinyue Peng, Na Zhang, Ying Liu, Hanxi Zhao, Ruiling Liu, Liyun Zhang, Ke Xu

**Affiliations:** 1 Department of Rheumatology, Third Hospital of Shanxi Medical University, Shanxi Bethune Hospital, Shanxi Academy of Medical Sciences, Tongji Shanxi Hospital, Taiyuan, 030032 China; 2 Department of Immunology, Shanxi Medical University, Taiyuan, 030000 Shanxi China; 3 Silc Business School, Shanghai University, Shanghai, 200444 China

**Keywords:** Stem cell, Senescence, Aging, Perception associated secret types, Epigenetics

## Abstract

Stem cells have self-renewal ability and multi-directional differentiation potential. They have tissue repair capabilities and are essential for maintaining the tissue homeostasis. The depletion of stem cells is closely related to the occurrence of body aging and aging-related diseases. Therefore, revealing the molecular mechanisms of stem cell aging will set new directions for the therapeutic application of stem cells, the study of aging mechanisms, and the prevention and treatment of aging-related diseases. This review comprehensively describes the molecular mechanisms related to stem cell aging and provides the basis for further investigations aimed at developing new anti-stem cell aging strategies and promoting the clinical application of stem cells.

## Background

Adult stem cells are undifferentiated cells residing in differentiated tissues in the adult body. Stem cells have multi-directional differentiation potential and self-renewal ability. They mediate tissue growth and regeneration under normal physiological conditions or in response to injury.

Adult stem cells exist in various tissues and organs of the body, including hematopoietic stem cells (HSCs), mesenchymal stem cells (MSCs), neural stem cells (NSCs), liver stem cells (LSCs), muscle satellite cells (MuSCs), skin epidermal stem cells (SESCs), intestinal epithelial stem cells (IESCs), retinal stem cells (RSCs), germline stem cells (GSCs), and pancreatic stem cells (PSCs). In scientific investigations, the MSCs are the most widely used owing to their wide sources, including bone marrow, fat, muscle, amniotic fluid, and cord blood. MSCs are a type of adult stem cells with a highly efficient self-renewal and multi-differentiation potential. They are easy to culture in vitro, can easily establish contact with host cells, and can display immune tolerance. They are ideal seed cells for regenerative tissue engineering and treatment of various diseases [1]. Adult stem cells have a variety of common features: they express telomerase for self-renewal and switch phenotypes between static/active states; their chromatin exists in two states for renewal or differentiation; they have unique metabolic requirements; and their intracellular macromolecular substances are distributed asymmetrically and cause asymmetric cell division [2].

Cellular senescence is a hallmark of aging, characterized by irreversible cell cycle arrest in response to various stress stimuli. Aging itself is an inevitable process affecting the genome, the cell, and the entire organism. It represents a major risk factor for many diseases, such as cancer and cardiovascular diseases. Accumulation of DNA damages, imperfect protein homeostasis, altered cellular communication, and exhaustion of stem cells are the main features of aging.

In general, when the number of cell divisions reaches the Hayflick limit, cells age and eventually die through apoptosis. In many different tissues, adult stem cells are responsible for these processes, replenishing dying cells to maintain normal tissue function and regenerating injured tissues. Hence, adult stem cells play a vital role in preventing the aging of organs and tissues, thereby delaying aging. However, during aging, the adult stem cells can undergo cellular senescence and apoptosis. Indeed, during the expansion and culture process, adult stem cells show senescence-specific phenotypes, such as morphological changes, decreased proliferation activity, and unbalanced biological functions.

Investigating the mechanism of stem cell aging is essential for the in vitro expansion of stem cells that can be employed in both basic and clinical research. Similarly understanding the stem cell aging will widen our understanding of organ aging.

### Cellular Senescence

Cellular senescence is the irreversible arrest of the cell cycle and inhibition of cell apoptosis. The activation of the p53 and p16^INK4A^/Rb tumor suppressor pathway underlies replicative senescence. Increasing evidence shows that cellular senescence is one of the causes of stem cell senescence [3]. In HSCs, satellite cells, and NSCs, p16^INK4A^, a marker of cellular senescence, accumulates with age, and inhibition of P16^INK4A^ can help improve the function of aging stem cells [4, 5]. As stem cells have the ability to self-renew and differentiate, inducing stem cells to senescence will obviously obliterate their functions. Aging cells can also regulate the functions of neighboring stem cells through the secretion of different senescence-associated secretory phenotypes (SASPs), whose expression is regulated by the activation of specific transcription factors, such as C/EBPβ, GATA4, NF-κB, mTOR, p38MAPK, Notch1 signaling molecules, MLL1 (KMT2A), HMGB2, H2A.J, and MacroH2A [6]. SASPs, including a series of inflammatory cytokines, chemokines, growth factors, and matrix metalloproteinases, can spread senescence to the surrounding and distant non-senescent cells through autocrine and paracrine mechanisms. Senescence is thus transmitted to adjacent cells, affecting the cell microenvironment and leading to sustained low-level chronic inflammation. When compared with proliferation arrest alone, the paracrine influence better explains the involvement of cellular senescence in various physiological and pathological processes. Different aging stimuli, cell types, and microenvironments induce distinct types and quantities of SASP factors. The results of single-cell RNA sequencing further showed that there were significant differences in the expression of SASP factors among different cells. SASPs can trigger the proliferation, angiogenesis, or epithelial-mesenchymal transition (EMT) of neighboring or cancer cells. SASPs include inflammatory interleukins (IL), such as IL-1, IL-6 and IL-8. The IL-1 signaling pathway is related to in vivo paracrine senescence after the activation of the inflammasome complex [7], whereas IL-6 and IL-8 can promote the development of cellular senescence, and their depletion prevents sentence in vitro [8]. Chronic stimulation of Bone marrow MSC (BMMSCs) with anti-proliferative cytokines, such as interferon β (IFNβ) and transforming growth factor β (TGFβ) can induce ROS-mediated p53 or p16^INK4A^ dependent senescence [9, 10]. The monocyte chemotactic factor-1 (MCP-1/CCL2), secreted by the MSCs derived from senescent human cord blood, can induce cellular senescence in vitro and in vivo by binding to its homologous receptor CCR2. SASPs can mediate immune cell activity to eliminate senescent cells, especially macrophages and natural killer cells. Therefore, local injection of senescent cells may be a new regeneration method to promote tissue function repair in the body.

Extracellular vesicles (EVs) are abundantly secreted by aged and senescence-induced cells. EVs include exosomes, microvesicles, and apoptotic bodies, which mediate material exchange and communication between cells. EVs can affect the function and fate of target cells through endocytosis, membrane infusion, and ligand-receptor interactions. Exosomes are small EVs with a diameter of 50 nm–150 nm, which contain specific components, such as proteins, lipids, DNA, RNA, and especially microRNAs (miRNAs or miR). The p53/TSAP6 axis seems to promote the transport of the endosomal system and exosomes/MVB and regulate the production of exosomes in senescent cells [11]. The increased rate at which senescent cells secrete exosomes may be a way to remove unwanted, toxic, and misfolded molecules and keep the cells safe. The exosomal content of senescent cells is different from that of normal cells. Interestingly, the exosomes constitute part of the SASPs and can mediate paracrine signaling effects on the microenvironment.

With aging, MSCs abundantly release exosomes with altered cargoes, such as miRNAs. MiRNAs are small single-stranded noncoding RNA molecules (about 22 nucleotides) that play a critical role in RNA silencing and post-transcriptional regulation of gene expression [12]. Different miRNAs are present in exosomes derived from either MSCs or senescent MSCs. Moreover, exosomes isolated from aged bone marrow contain high levels of miR-183 cluster and miR-31a-5p, which reduce MSC proliferation and osteogenic differentiation, promote MSC aging, and stimulate both osteoclastogenesis and bone resorption [13]. In addition, miR-17, miR-34, miR-146a, miR-183 p-5p, and miR-335 p-5p were upregulated in senescent MSCs [14]. These miRNAs are mainly involved in inhibiting pro-apoptotic genes and regulate processes such as cell senescence, reduction in the number of stem cells, telomere erosion, and circadian rhythm, controlling the decline of tissue-specific adult stem cell functions during aging.

### Telomere and Telomerase

Telomeres are special structures that are located at the ends of linear chromosomes in eukaryotic cells and are composed of short and highly repetitive non-transcribed DNA sequences (TTAGGG) and structural proteins. They play an important role in chromosome positioning, replication, and protection, and, thus, in the regulation of cell growth and lifespan. As the number of cell divisions increases, telomeres gradually shorten. When telomeres are too short that DNA is unable to continue to replicate and chromosome stability is at risk, stem cell senescence is induced, resulting in cell division stagnation, inability to continue to replicate, and eventually apoptosis [15]. Therefore, telomere shortening is a common sign of senescence in all cells, including stem cells.

The length of telomeres is maintained by the action of a specific enzyme called telomerase (a reverse transcriptase). Telomerase can replace errors during DNA replication to repair and extend telomeres. Telomerase can prevent telomeres from being lost because of cell division. However, during aging, although stem cells express telomerase, the telomeres of HSCs, NSCs, HFSCs, and GSCs would still shorten [16]. When overexpressing telomerase reverse transcriptase (telomerase catalytic subunit; TERT) in mouse models for either anti-tumor therapy or advanced age, their median survival time increased independently from the tumor incidence, indicating that telomere length contributes to survival during aging [17]. However, the correlation between increased survival and stem cell activity remains unclear. Clinical studies showed that telomere length is negatively correlated with age in people older than 75 years old, and longer telomeres promote the survival of elderly individuals [3]. Interestingly, when compared with the telomeres of mouse MSCs, human MSCs have significantly shorter telomeres and will not spontaneously initiate transformation during long-term culture to escape cell senescence. Down-regulation of human telomerase in MSCs can shorten telomere length, inhibit cell proliferation, and promote cell apoptosis, whereas overexpression of human telomerase can increase telomere length, promote cell proliferation, and reduce cell apoptosis [18]. In addition, studies have found that telomere integrity depends not only on the telomere length but also on the epigenetic status of telomere/subtelomere regions. GADD45a induces the demethylation of CpG islands that are dependent on base excision repair to produce a permissible chromatin state for DNA damage response (DDR), especially in the short telomere/subtelomere regions. Depletion of GADD45a promotes chromatin condensation in the subtelomere regions. GADD45a knockout can improve the function of intestinal stem cells and extend the lifespan of telomerase-deficient mice (G3Terc−/−) [15].

### DNA Damage and Mutations

The mutation accumulation theory is one of the earliest senescence theories, supporting that DNA damage and mutation accumulation are involved in the process of stem cell senescence. In HSCs, histone H2AX phosphorylation and comet tail are signs of DNA damage, both of which increase with age [19, 20]. In satellite cells, H2AX phosphorylation also accumulates with age [21]. Aging HSCs can also show replication stress and decreased expression of DNA helicase, making it easier to face challenges in subsequent replication [22]. The lifetime risk of cancer in tissues is related to the number of divisions of stem cells. Every time a stem cell copies DNA and divides, the possibility of carcinogenic transformation increases. Many kinds of stem cells, including HSCs and satellite cells, are mostly in a quiescent state. This represents a protection mechanism against acquired replication damage; however, acquired mutations are more likely to occur when DNA is damaged because double-strand breaks in quiescent stem cells are more likely to be repaired by error-prone non-homologous end joining methods [23], whereas proliferating stem cell employ a more accurate repair system, the homologous recombination [19]. HFSCs also use non-homologous end repair after radiation exposure similar to that followed by the other types of skin cells [24]. Therefore, although proliferating stem cells are more susceptible to DNA damage, their repair system is more precise than that of stem cells in a resting state.

The effects of DNA damage on stem cells during aging include: (1) accumulation of mutations that promote proliferation and survival. Several studies have found that the number of HSCs with pre-cancerous mutations increases during senescence [25–27]; and (2) induction of apoptosis, senescence, or differentiation, which leads to a reduction in the number of stem cells. Studies have found that mice deficient in DNA damage repair exhibit premature aging [28]. Moreover, upregulation of the expression of silent information regulatory protein 6 (SIRT6) to enhance DNA repair can extend their lifespan [29]. However, the direct association between increased lifespan and increased stem cell survival remains unclear.

DDR causes high genome instability and promotes stem cell senescence. When cells undergo DDR and the rapid repair mechanism fails to repair the damage, it will lead to cell senescence and, potentially, to apoptosis. Studies in *Drosophila* showed that depletion of DDR-related genes, such as *ATM, ATR, Chk1*, and *Chk2* in the intestinal cells, reduced DDR and promoted cell death and cellular senescence, ultimately affecting the lifepan of *Drosophila* [30]. Cumulative DNA damage can induce endogenous IFNβ, amplify the DDR through related signal pathways, activate p53 signal pathway, shorten telomere length, and promote MSCs senescence [31]. The age-related loss of heterochromatin stability in the intestinal cells of *Drosophila* is closely related to the senescence of intestinal stem cells. This phenotype is associated with the decreased function of H3K9me3 and heterochromatin protein 1 (HP1) that leads to genomic oxidative stress and activation of AKT/TOR signaling pathway [32].

### Epigenetic Alterations

Epigenetic regulation can affect the accessibility and plasticity of the genome, which is important for the process of aging. Epigenetic regulation includes molecular processes, such as DNA methylation, histone modification, chromatin remodeling, and non-coding RNAs. In aging HSCs, an increase in the expression of gene clusters at different positions of the chromosomes is observed, indicating that epigenetic disorders can cause the loss of regionalization of transcriptional silencing. These findings suggest that epigenetic modification is a common feature of stem cell aging and that it affects stem cell’s functions.

#### DNA Methylation

Regulation of chromatin state plays an important role in the stem cell function. The chromatin is found in two states: active, also known as open chromatin or euchromatin, and inactive, also known as heterochromatin. Changes in chromatin and gene expression have been observed during the aging process. In aging HSCs, the expression of gene clusters in different chromosomal loci can increase, indicating that epigenetic imbalance can cause the loss of regionalization of transcriptional silencing.

DNA methylation is the selective addition of a methyl group to specific regions of the genomic, called CpG islands. CpG islands are often located in the promoter region of genes and have a critical role in the regulation of regulating gene expression, transposon silencing, variable splicing, and genomic stability. Studies have found that in aging HSCs the DNA methylation level in the open chromatin state is upregulated in lymphoid cells, whereas it is downregulated in myeloid cells [33]. The target genes of Polycomb-group proteins (PcGs) show hypermethylation during aging. In DBMT3a/b knockout mouse embryonic stem cells, PcGs tend to bind to unmethylated CpG islands to regulate methylation levels [34]. PcGs are recruited to the unmethylated DNA by the Polycomb repressive complex protein, KDM2B [35].

In a study investigating the transplantation of DNMT3a/3b deficient HSCs, it was found that the differentiation ability of these defective HSCs to myeloid and lymphoid cells remained unchanged after transplantation, whereas their continuous renewal ability decreased. This finding proved that DNMT3a/b could protect the self-renewal ability of HSCs [36]. In DNMT3a knockout NSCs, neurogenesis related genes, such as Dlx2, Sp8 and Neurog2, were downregulated. This phenotype was positively correlated with the increase in H3K27me3 level, revealing that DNA methylation and histone methylation modification can synergistically regulate the expression of target genes [37].

#### Histone Modification

Histone modification affects the amino acids of the histones and includes histone acetylation, methylation, phosphorylation, and ubiquitination. These modifications change the accessibility to the chromatin, thus regulating gene expression. The processes of histone methylation and acetylation are linked with to aging.

Histone methylation modification generally refers to methylation post-translational modification of histone lysine residues, which are regulated by histone methyltransferase. In general, H3K4me3 and H3K27me3 play a promoting or inhibitory role, respectively, in transcriptional regulation and contribute to the regulation of lifespan-related genes in several models [38–40]. Aging HSCs show high level of lysine trimethylation at position 4 of histone H3 (H3K4me3) in chromatin regions associated with self-renewal, potentially leading to an increase in the number of HSCs [41]. In aging satellite cells, the level of H3K4me3 decreased slightly, whereas lysine trimethylation at position 27 of histone H3 (H3K27me3) increased significantly [42].

In the premature aging model of human adult stem cells induced by the nuclear membrane protein laminA/C gene mutation, a downregulation of the histone methyltransferase EZH2 was observed. Moreover, a reduction of lysine trimethylation at position 9 of the whole histone and its binding protein HP1α and a loss of heterochromatin were also detected [43]. EZH2 overexpression promotes HSCs long-term regeneration ability [31]. In addition, studies have shown that the methyltransferase Dot1/DOT1L catalyzes the methylation of histone H3 lysine 79 (H3K79), which is associated with the silencing of telomere regions, development, cell proliferation checkpoint, DNA repair, gene transcription [44].

Histone acetylation modification refers to the modification of the N-terminal lysine residues of H3 and H4, catalyzed by histone acetyltransferases (HAT) and histone deacetylases (HDACs). Histone acetylation modification can activate gene transcription by loosening the nucleosome structure and exposing the DNA sequence specifically bound by transcription factors. Class III HDACs, also known as sirtuins (SIRTs), are associated with aging [45]. Human sirtuin family proteins homologous to yeast Sir2 are essential for maintaining the homeostasis of adult stem cells during aging. Both SIRT6 and SIRT7 proteins belong to the NAD + - dependent histone deacetylase.

Sirt7 deletion causes premature aging and shortens the lifespan in mice [46, 47], suggesting that SIRT6/7 has potential anti-aging effect. Human MSCs with homozygous depletion of SIRT6 show characteristics of accelerated aging [48]. SIRT6 interacts with Nrf2 and deacetylates H3K56, thus recruiting the RNA polymerase II complex, activated Nrf2 transcription. SIRT6-HSCs show an increase in the level of overall histone H3K56ac, which is unable to recruit RNA polymerase II to bind to the promoter region of Nrf2, promoting the aging phenotype [49, 50]. In addition, in vitro experiments have also shown that SIRT1 knockdown in human retinal stem cells promoted premature aging phenotype. Upon SIRT1 overexpression, this premature aging phenotype can be rescued [51]. Furthermore, it was found in a mouse model that the activation of Cdc42 activity inhibited the expression of nuclear membrane protein laminA/C protein, reduced the distribution of H3K16ac, affected the chromosomal state, and caused the aging of HSCs [52]. Moreover, aging HSCs show a reduction in acetylation modification of lysine residue at position 16 of histone H4 (H4K16ac) level. In transplantation experiments, inhibition of CDC42 can restore H4K16ac to the level of young HSCs, thus reversing its senescence phenotype [53].

#### Chromatin Remodeling

Chromatin remodeling refers to the change in chromatin packaging state, histones localization in nucleosomes, and corresponding DNA molecules. An increasing number of studies has shown that the function of chromatin remodeling regulatory proteins is closely related to the process of aging. Several chromatin structure changes occur in the adult stem cell models of both physiological aging and premature aging [54] and are associated with increased DNA damage and dysregulation of BRG1, a component of the ATP-dependent chromatin remodeling complex, which is related to the senescence of MSCs, by regulating the methylation status of NANOG [55].

Cell chromatin gradually undergoes permanent changes when DNA is damaged, and the type of damage that has the greatest effect on chromatin is DNA double-strand break. Therefore, chromatin remodeling caused by DNA damage may form the basis of the distorted cell phenotype of senescent stem cells [56]. Whether long-lived animals or humans can relatively resist the epigenetic changes caused by DNA damage and whether inducing DNA double-strand breaks in stem cells will accelerate their aging, remain to be further clarified. The change in chromatin status may affect the expression of aging-related genes. Further research is needed to explore the mechanism of chromatin remodeling proteins regulating stem cell aging and ways to therapeutically target this process to achieve healthy aging and even reverse aging.

### Non-coding RNA

Non-coding RNAs refer to RNA molecules that do not encode proteins and include ribosomal RNA, transport RNA, small nuclear RNA, miRNA, and long non-coding RNA (lncRNA). These molecules regulate post-transcriptional processes mediating the expression of genes.

#### miRNAs

Intracellular miRNAs play a role in the steady-state regulation of stem cells fate. Moreover, as previously mentioned, miRNAs-containing exosomes can be used as SASPs to regulate cell senescence. For example, in skeletal muscle, MuSCs are in a static state. In mice, the MuSCs activation rate between different muscles depends on the level of the transcription factor Pax3. Pax3 is regulated by miR206. Another miRNA regulating cellular senescence in NSCs and MSCs is let-7, which mediates the downregulation of HMGA2. HMGA2 promotes adult stem cells self-renewal by decreasing the expression of p16^INK4A^ [57, 58]. The low expression of miR-34 in cancer can inhibit HDM4, which is a negative regulator of p53 [59]. In MSCs, the expression level of miR-34a increased with each cell passage [60]. MiR-34a induces aging of bone marrow mesenchymal stem cells by downregulating the cell cycle regulators and the differentiation to osteoclasts and adipocytes. MiR-29 inhibits tumor growth by increasing p53 expression, activates the p53-p21 and p16 PRB pathways via regulation of CNOT6, and impairs the proliferation of muscle progenitor cells in elderly mice by binding to the 3’-UTR of p85a, IGF-1, and B-Myb [61, 62]. Aging human BMMSCs showed progressive expression of mir-29 [63]. Mir-132 cluster regulates HSC aging and is highly expressed in long-term HSCs [64]. The miRNA-targeting transcription factor FoxO3 (an important regulator of aging) can promote hematopoiesis. Mir-132 leads to FoxO3 dependent changes in HSC autophagy and viability. MiR-146 can inhibit NF-κB, acting as a negative regulator of cancer metastasis [65]. Depletion of miR-146a leads to a decrease in the number of HSCs [66]. After depletion of miR-146a, HSCs lose the in vivo hematopoietic potential. High expression of miR-195 in human BMMSCs from elderly donors shortens the length of telomeres [67]. MiR-195 directly targets the 3’-UTR of the TERT gene. Knockdown of MiR-195 stabilizes telomere length and improves the therapeutic effect of heart repair and the proliferation of BMMSCs. Another miRNA up-regulated in human BMMSCs from aging donors is miR-196. HOXB7 is a direct target of miR-196. Overexpression of HOXB7 increases the proliferation and osteogenic differentiation of human bone MSCs [68]. Mir-141 p-3p expression is dependent on histone acetylation on the cell surface and increases during the aging of human MSCs. Mir-141 p-3p and its direct target CDC25A lead to G1 cell cycle arrest and impaired osteogenic differentiation of human MSCs [69]. The increase of miR-335 in elderly BMMSCs induced by elderly donors [70] further disables the differentiation ability and immunomodulatory properties of chondrocytes. MiR-335, indeed, targets AP-1, which regulates cell migration, differentiation, and proliferation. In human adipose tissue-derived bone MSCs, mir-486 p-5p is overexpressed with replicative aging, leading to premature aging [71]. MiR-486 p-5p plays an inhibitory role by binding to the 3’-UTR of SIRT1 gene, which in turn plays an important role in mammalian cell aging. MiR-543 and mir-590 p-3p regulate the cellular senescence of human MSCs by inhibiting the AIMP3/P18 pathway [72]. The expression level of AIMP3/P18 protein in aging BMMSCs increases, regulating the senescence and differentiation potential of MSCs. Mir-543 and mir-590 p-3p significantly reduce the expression of AIMP3/P18, and their overexpression inhibits the aging of late passage MSCs.

#### lncRNA

lncRNA-BMNCR is a key regulator of age-related osteogenic niche changes and cell fate transition in BMMSCs. BMNCR regulates the osteogenic niche of BMSCs by maintaining the extracellular matrix protein fibromodulin and activating the BMP2 pathway. Restoring BMNCR levels in human BMMSCs can reverse the age-related transition between osteoblast and adipocyte differentiation [73]. Lncrna-ZEB2-NAT is the natural antisense chain of Zeb2, which controls the expression of zinc finger E-box-binding homebox 2 (ZEB2). ZEB2-NAT regulates the expression of ZEB2 in mouse fibroblasts and embryonic stem cells. Reducing the level of ZEB2-NAT RNA leads to the production of totipotent stem cells induced by the transient transcription factor (OSKM). In addition, it was also found that when embryonic stem cells receive signals, ZEB2-NAT expression is rapidly upregulated and ZEB2 expression is blocked, thus affecting the self-renewal and pluripotency of embryonic stem cells [74]. Luo et al. compared the expression of lncRNAs between HSCs of different ages and those between wildtype (wt) and DNMT3a-deficient mice-derived HSCs (DNMT3a knockout). The latter showed defective differentiation [75]. LncHC-1 and lncHC-2 were found to be highly expressed in wt HSCs and missing in DNMT3a knockout HSCs. Surprisingly, the lncRNA whose expression changes with age still remains unknown [76]. Silencing of SPEHD, a mouse lncRNA, in myeloid progenitor cells leads to dysfunction in the oxidative phosphorylation pathway [77].

MSCs isolated from aged mice showed decreased cell proliferation, increased reactive oxygen species (ROS), and increased lincRNA-p21 levels. In contrast, silencing of lincRNA-p21 enhances cell growth [78].

The HOX Transcript Antisense RNA (HOTAIR) is involved in the regulation of MSC proliferation and differentiation. Interestingly, the expression level of HOTAIR remains unchanged with aging in vitro, but its regulation affects the adipogenic differentiation ability of MSCs, as HOTAIR is related to the changes of aging related gene expression and DNA methylation profile [79].

### SIRT Family of Proteins

SIRT is a class of evolutionary conserved histone deacetylase and ADP-ribosyltransferase proteases, which are involved in the regulation of inflammation, energy metabolism, and aging processes. SIRT1 plays an important role in maintaining the proliferation and differentiation of MCSs and in regulating aging. BMMSCs with selective SIRT1 knockout showed that the cell growth slowed down in the early stage, gradually entered the cell stagnation phase, and the cell aging accelerated. At the same time, the number of cells in S phase decreased significantly, and the expression of p16 and p21 decreased, while SIRT1 gene knockout of adipose tissue-derived MSCs could increase the expression of p16 and p21 [56]. High glucose can increase the expression of miR-486 in adipose MSCs, inhibit the expression of SIRT1, and promote cell senescence [71]. Resveratrol can regulate the self-renewal ability and differentiation ability of umbilical cord-derived MSCs through the SIRT1 signaling pathway in a dose-dependent manner. It can increase the viability and proliferation ability of MSCs, reduce cell senescence, and inhibit the expression of p53 and p16 [80]. The mechanism is related to nicotinamide phosphoribosyltransferase, which is the rate-limiting enzyme for NAD+ synthesis. Restoring mitochondrial NAD+ levels can delay senescence during MSCs replication and prolong the lifespan of MSCs [81]. In addition to SIRT1 in the SIRT family, SIRT2, SIRT3, and SIRT6 also have anti-aging functions. Overexpression of SIRT3 activates the expression of superoxide dismutase (SOD) and catalase, thereby improving the damage to MSCs caused by oxidative stress and reducing cell apoptosis. However, severe oxidative stress can lead to a decrease in SIRT3 in BMMSCs [82]. SIRT6 maintains genome stability by affecting the process of DNA damage repair, and its functional defects induce cell senescence. In BMMSCs, knocking out the SIRT6 gene can impair cell proliferation and migration and resistance to oxidative stress and accelerate cell senescence [83].

### Nutrient Sensing and Metabolism

The most effective intervention for prolonging life is to restrict calorie intake without malnutrition. One of the benefits of calorie restriction may generate from changing the phenotype of stem cells. Regulating energy metabolism pathways to reduce cell damage and maintain the niche of original stem cells may promote the normal function of stem cells and the homeostasis of tissues [84]. Calorie restriction increased the abundance of satellite cells in muscles and improved the functions of several stem cell populations in mice, including HSCs [85] and GSCs [86]. Calorie restriction also promoted the self-renewal of intestinal stem cell (ISC) in mice by inducing Paneth cells, which constitute the microenvironment of stem cells, to express the BST1 enzyme [87]. Insulin-like IGF signal, TOR signal, AMPK, sirtuins, and FOXO transcription factors are pathways or factors of calorie restriction-mediated longevity, suggesting that they are involved in the response of stem cells to calorie restriction [88].

Metabolic state and ROS also play an important role in stem cell senescence. Oxidative stress damage caused by pathological conditions produces a large amount of ROS, which has cytotoxic ability and can induce cell damage. With the increase of age and the number of passages in vitro, the ROS produced by MSCs gradually increase. Excessive ROS or exogenous H_2_O_2_ can damage the proliferation and differentiation ability of MSCs. Sublethal amounts of ROS and ionizing radiation can damage the DNA of human umbilical cord-derived MSCs, decrease DNA synthesis, slowdown cell proliferation, and, finally, cause cell senescence. Exogenous H_2_O_2_ induces the senescence of human endometrial-derived MSCs. However, inhibiting the p38/MAPK signaling pathway can partially alleviate the cell senescence induced by H_2_O_2_ [89]. The sublethal dose of H_2_O_2_ can arrest the cell cycle of BMMSCs in G0/G1 phase and significantly inhibit the osteogenic differentiation of cells. The antioxidant melatonin promotes the proliferating cells to enter the S phase in a concentration-dependent manner and inhibits the p38/MAPK signaling pathway, thereby reducing the senescence of MSCs [90]. High concentrations of ROS accelerate MSC senescence by promoting defective angiogenesis [91]. Adding lycopene to the culture medium of senescent MSC can significantly reduce the level of ROS through the P13K/AKT signaling pathway and delay the senescence of MSCs. Monocyte chemoattractant protein-1 (MCP-1) is an important component of the senescence-related secretory phenotype of MSCs cultured in vitro. MCP-1 acts on chemokine receptor 2, activates the ROS-p38-MAPK-p53/p21 signaling pathway, prevents the phosphorylation and degradation of RB protein, and promotes MSC senescence [92].

Static stem cells usually rely on glycolysis to produce energy, which can reduce the abundance of ROS [93, 94]. A variety of adult stem cells also exist in a hypoxic environment, which can further limit the production of ROS [95]. The proliferation of stem cells is highly dependent on oxidative phosphorylation, which is prone to oxidative damage and cell dysfunction. Elimination of ROS molecules or overexpression of the transcription factor Nrf2, which regulates oxidative stress response, can help reduce the hyperproliferative phenotype of aging ISCs [96, 97].

The reduction in the number of mitochondria in stem cells may also cause senescence-related changes in stem cell function [98]. The PGC-1 homologous gene, spargel, is a favorable regulator of mitochondrial biology. When overexpressed in *Drosophila* ISCs, it can effectively inhibit ISC senescence and prolong average lifespan [99]. HSCs and satellite cells can increase glucose and glutamine metabolism during the activation process [100, 101]. This is a way to efficiently use sugars to produce ATP, which mimics the Warburg effect of tumor cells. Similarly, skeletal muscle is accompanied by pseudo-hypoxia and Warburg-like metabolism during aging, which damages cell function [102] and promotes malignant transformation [103].

### Cell Polarity and Proteostasis

Stem cells can use different mechanisms to prevent the accumulation of damage components, such as asymmetric isolation of damaged proteins, and enhancement of protein homeostasis. Stem cells can either divide symmetrically to produce two daughter cells with the same fate, or divide asymmetrically to produce a daughter stem cell and a differentiated cell. In the process of asymmetric division, the distribution of cell components is not uniform. Considering that progeny stem cells may survive longer than differentiated cells, undamaged components will be enriched in progeny stem cells. Stem cells can also asymmetrically isolate damaged proteins and mitochondria [104–106]. This asymmetric division requires cell polarization and the ability of senescent GSCs and HSCs to polarize division decreases, indicating that loss of polarity can contribute to stem cell senescence [107]. Changes in the Wnt signaling pathway associated with aging in HSCs result in loss of polarity [108]. Stem cells can also eliminate damaged proteins by maintaining high levels of autophagy and proteasome activity. For example, HSCs and skin stem cells have stronger autophagy ability than peripherally differentiated cells [109]. Although it has not been determined whether adult stem cells have proteasome activity, human embryonic stem cells have shown high proteasome activity [110]. In addition, stem cells activate autophagy to eliminate cellular waste generated during the resting phase. Two independent studies on muscle and hematopoietic stem cells have demonstrated the correlation between autophagy damage and stem cell depletion and aging [111].

### Niche Deterioration

Stem cell niche is a microenvironment around the stem cells that plays a critical role in maintaining the stemness properties and proper function of the stem cells. The aging of stem cell microenvironment can also significantly affect the function of stem cells. Allogeneic transplantation and conjoined experimental studies have shown that the aging of satellite cells, NSCs and GSCs is mainly driven by exogenous mechanisms. In experiments involving *Drosophila*, the number of stem cells that form the survival microenvironment decreased significantly due to a decrease in the self-renewal ability of stem cells after aging [112, 113] and in the number of factors that regulate the adhesion of GSCs to the microenvironment [112, 114].

After aging the supporting cells present in the survival microenvironment are unable to send appropriate morphogenesis and growth factor signals to the stem cells, thus affecting the direction of cell fate. For example, fibroblast growth factor (FGF) can reduce the expression of MDM2 through the P13K/AKT signaling pathway. The latter acts as a key negative regulator of p53 and inhibits cell senescence by regulating the expression of Cyclin G [115]. The expression of FGF2 in the satellite cell microenvironment of mouse muscles increases after aging and can impair the self-renewal of stem cells [116]. The microenvironment of patients with systemic lupus erythematosus contains high levels of Leptin and NAP-2, which can induce MSCs to age. Treatment with the P13K/AKT inhibitor LY294002 significantly reduces the effects of Leptin and NAP-2 [117]. Inflammatory markers also increase after aging in the survival microenvironment of stem cells and can negatively affect stem cell function [118]. Therefore, increased inflammation may be considered a marker of aging. In addition, the time regulation of adult stem cell physiology mediated by the circadian clock is essential for maintaining the homeostasis of body tissues and stem cells. The regulation of the transcription/translation system and the circadian rhythm need adult stem cell microenvironment to maintain a specific homeostasis [119]. Furthermore, the concentration of glucose in the internal and external microenvironment significantly affects cell proliferation, differentiation, senescence, and apoptosis. The high glucose environment can increase the expression of miR-486 in adipose MSCs, inhibit the expression of SIRT1, and promote cell senescence [71]. The SIRT1 inhibitor resveratrol can protect MCSs from senescence induced by a high-sugar environment [120]. High glucose-induced protein O-nitroacetylglucosamine (O-GlcNAc) glycosylation modification can inhibit cell proliferation, block cell cycle, and promote cell apoptosis and senescence [121].

Another major exogenous factor that contributes to stem cell senescence is the change in the concentration of circulating factors. The discovery of these factors derives from the observation that blood or serum extracted from young or calorie-limited animals can rejuvenate senescent cells. Insulin and insulin-like growth factor-1 are important factors among them. These molecular signals mediate the lifepan-increasing effect of calorie restriction on mice to a large extent, which was evident from previous studies wherein it was observed that the circulating IGF-1 levels in the growth hormone receptor knockout mice were very low and many types of calorie restrictions were unable to bring additional life-enhancing benefits [122, 123]. Specifically blocking the insulin-IGF signal in *Drosophila* ISCs can improve the homeostasis of the intestinal environment and prolong lifespan. The decrease of IGF-1 level is related to the improvement of HSCs self-renewal [124, 125]. At the same time, studies have found that insulin, IGF-1, and growth hormone can increase the number of NSCs and GSCs in aging animals [126–128], suggesting that NSCs or GSCs may increase their numbers through the above cyclic factors at the expense of long-term self-renewal or affect the steady-state process. TGF-β is also a cyclic factor that regulates stem cell function during aging. TGF-β levels increase in the serum of aging mice or humans, which in turn affects the function of satellite cells and NSCs [129, 130]. Growth differentiation factor 11 can improve the function of satellite cells and NSCs, and its level will also decrease after aging [131, 132].

## Conclusion

**Fig. 1:**
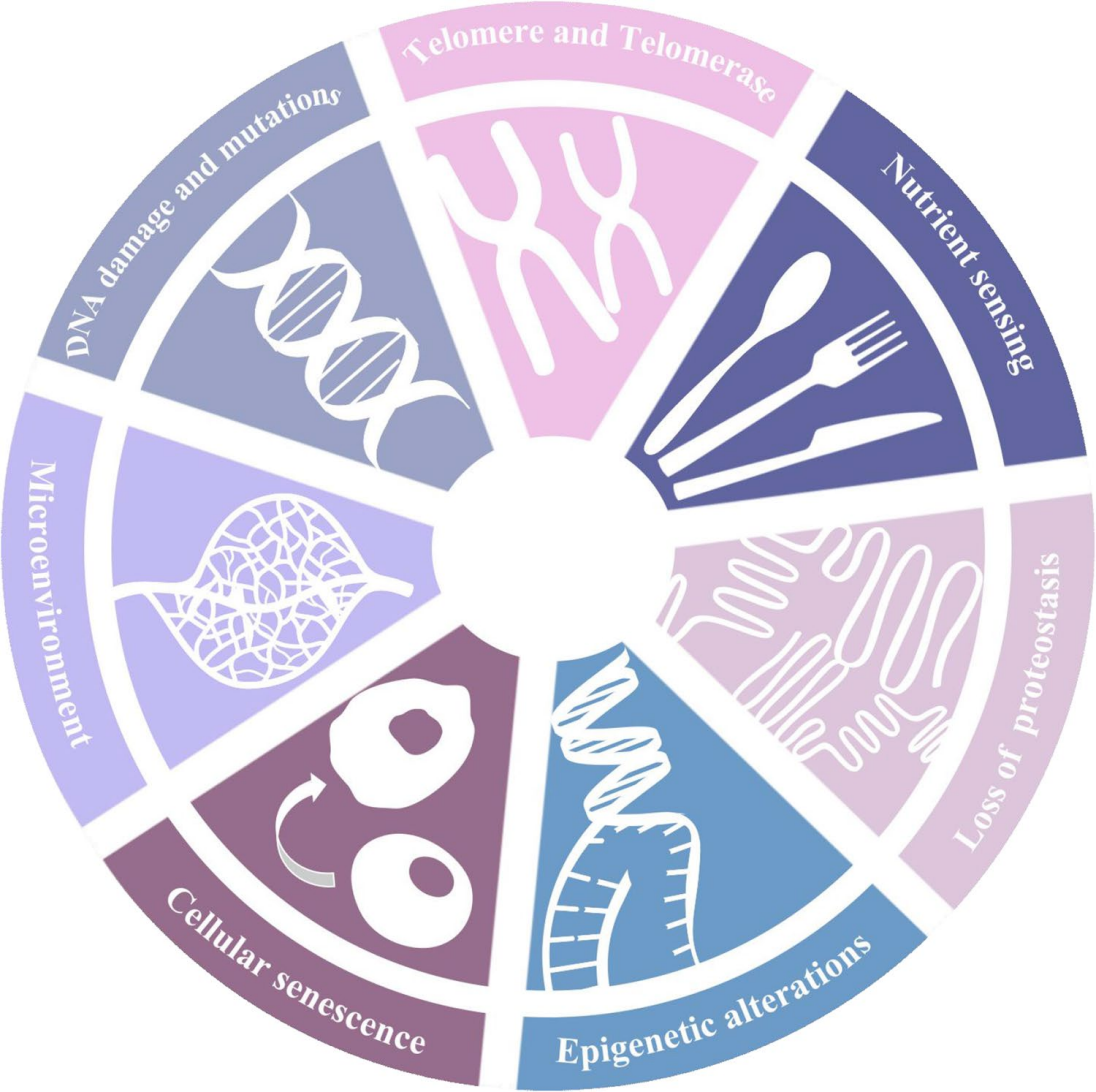
The Hallmarks of stem cell senescence. The scheme enumerates the seven hallmarks described in this Review: cellular senescence, DNA damag and muations, telomere and Telomerase, epigenetic alterations, microenvironment, deregulated nutrient sensing and cell polarity and proteostasis

In summary, stem cells of the body can maintain the continuous renewal of somatic cells, and their senescence may be the root cause of the aging in life. Therefore, delaying the aging of stem cells is an important breakthrough in the anti-aging research. Although the current understanding of stem cell aging has made some progress (Fig. 1), our understanding of the mechanisms underlying aging is limited. We believe that with the continuous deepening of stem cell aging research and the continuous development of anti-aging strategies, the clinical application of stem cells delaying human aging would gradually come to fruition.

## Data Availability

Not applicable.
